# Frailty in the anti-amyloid era: an opportunity to individualize care

**DOI:** 10.1016/j.tjpad.2026.100682

**Published:** 2026-10-09

**Authors:** Nicholas M. Vogt, Dae Hyun Kim

**Affiliations:** a Hinda and Arthur Marcus Institute for Aging Research, Hebrew SeniorLife, Boston, MA, USA; b Harvard Medical School, Boston, MA, USA; c Division of Gerontology, Department of Medicine, Beth Israel Deaconess Medical Center, Boston, MA, USA

The arrival of anti-amyloid therapies has fundamentally changed the clinical landscape of Alzheimer’s disease (AD). Around the world, as regulatory approval expands for lecanemab and donanemab, memory clinics and health systems are rapidly building the infrastructure to deliver these new treatments. This requires new clinical pathways to identify individuals with biomarker-confirmed early symptomatic AD and to assess treatment eligibility and contraindications. Treatment itself depends on close coordination between clinicians, infusion facilities, and radiology services, with ongoing clinical and MRI monitoring to detect amyloid-related imaging abnormalities (ARIA) and manage other complications. As access to anti-amyloid therapies expands and more patients are evaluated for treatment, the population entering these clinical pathways will inevitably be broader and more varied than the participants enrolled in the pivotal clinical trials. Given that age remains the strongest risk factor for AD, these systems must therefore also account for the health and functional challenges that become increasingly common with aging. Frailty offers a well-established framework for systematically capturing this complexity.

Broadly, frailty reflects reduced physiologic reserve and increased vulnerability to health stressors. Frailty integrates information across multiple aspects of health to help distinguish individuals who may be the same chronological age but differ substantially in their ability to withstand illness, tolerate treatment, or recover from acute medical events. Importantly, frailty assessment can characterize risk and guide clinical care [1], with frailty-informed approaches improving outcomes among older adults undergoing surgery [2] or cancer treatment [3]. Frailty is particularly relevant to AD, where it is common among patients with symptomatic AD and associated with adverse clinical outcomes [4]. Yet frailty has not been systematically incorporated into anti-amyloid trials or treatment pathways. We therefore know surprisingly little about the frailty of participants in the clinical trials or of patients now being evaluated and treated in clinical practice.

In this issue of *Journal of Prevention of Alzheimer’s Disease*, Bellelli and colleagues take an important step toward addressing this gap by outlining how frailty assessment could be integrated into the care of AD patients being considered for disease-modifying therapy [5]. Drawing on a multidisciplinary panel of experts, the authors propose that frailty should serve not as an immediate contraindication to treatment, but as a “clinical compass” that complements biomarker and disease-specific information and helps place treatment decisions within the broader context of an individual patient’s health. Importantly, they move beyond advocating simply for frailty screening and propose a practical clinical pathway in which identification of pre-frailty or frailty prompts more comprehensive assessment and multidisciplinary discussion. This assessment may identify opportunities to address contributors to frailty before or alongside disease-modifying therapy, with reassessment as a patient’s health changes over time. By treating frailty as a dynamic and potentially modifiable feature of health rather than a fixed barrier to treatment, the framework offers a thoughtful approach to incorporating the complexity of older adults into traditional disease-specific treatment pathways.

Bellelli and colleagues offer a compelling vision for what frailty-informed AD care could look like. Translating that vision into clinical practice, however, raises several important questions. Perhaps the most fundamental is what, specifically, clinicians should do differently once frailty has been identified. As noted, frailty should not simply become another criterion for excluding older adults from treatment, but its value will ultimately depend on whether and how it changes care. For a patient identified as frail, several reasonable paths may follow: proceeding with treatment, first addressing contributors to frailty, or initiating treatment alongside interventions targeting frailty. The latter approaches also require considering how best to intervene on frailty itself in the context of anti-amyloid treatment. Frailty can be modified, but interventions will need to be tailored to the specific deficits contributing to an individual patient’s frailty. Yet we currently have little evidence to guide which approach is appropriate for which patients. Nor is it clear whether improvement or worsening of frailty should alter treatment decisions over time. Translating frailty assessment into better care will therefore require moving beyond identifying vulnerability to understanding when, and in what ways, that information should influence clinical management.

Answering these questions will require evidence that is currently largely absent. We do not yet know how frailty relates to the outcomes of anti-amyloid therapy, including treatment tolerability, adverse events like ARIA, treatment interruption or discontinuation, or longer-term cognitive and functional outcomes. Retrospective analyses of existing trial data may provide some initial insight where frailty can be reconstructed from available variables, but prospective measurement will be essential. Real-world treatment programs and registries provide an immediate opportunity to characterize frailty among the broader population now receiving these therapies and determine how it relates to treatment outcomes. Future clinical trials should similarly incorporate frailty assessments by design. Collecting these data now will be far easier than attempting to reconstruct them years later. These efforts will also require greater consistency in how frailty is measured: numerous instruments exist, and determining which tools are most informative and feasible in the context of AD treatment will be essential if findings are to be compared across studies and ultimately translated into clinical care.

These questions may become even more important as anti-amyloid therapy moves into the preclinical stage of AD. Three ongoing phase 3 trials (the AHEAD 3-45, TRAILBLAZER-ALZ 3, and PrevenTRON studies) are testing anti-amyloid therapies in cognitively unimpaired individuals with evidence of AD pathology. If successful, these studies would represent a major advance toward preventing or delaying cognitive impairment and could substantially change the impact of AD on patients and families. At the same time, success would raise a new set of questions about whom to treat in clinical practice. The prevalence of amyloid positivity among cognitively unimpaired individuals rises steeply with advancing age, meaning that many candidates for future prevention will be older adults with widely varying health and functional status. Yet all three trials capped enrollment at age 80, limiting what these studies will tell us about preventive treatment in the oldest adults. If these trials prove effective, frailty may offer a valuable framework for helping individualize preventive treatment decisions for this expanded population.

The development of effective disease-modifying treatments for AD represents extraordinary progress after decades in which treatment options were limited largely to symptomatic therapies. As health systems build the infrastructure needed to deliver these treatments, there is an opportunity to incorporate frailty assessment into emerging clinical pathways from the outset. Frailty can help clinicians individualize care by capturing differences among older adults that are not reflected by age, AD biomarkers, or cognitive status alone. An important next step for the field is ensuring that advances in disease-specific treatment are integrated with the broader health needs of the older adults who receive them.

## Declaration of generative AI and AI-assisted technologies in the writing process

No generative AI or AI-assisted technologies were used in the preparation of this manuscript.

## CRediT authorship contribution statement

**Nicholas M. Vogt:** Conceptualization, Writing – original draft. **Dae Hyun Kim:** Writing – review & editing.

## Declaration of competing interest

The authors declare that they have no known competing financial interests or personal relationships that could have appeared to influence the work reported in this paper.
